# Electrophysiological investigation of the contribution of attention to altered pain inhibition processes in patients with irritable bowel syndrome

**DOI:** 10.1186/s12576-020-00774-x

**Published:** 2020-10-06

**Authors:** Nabi Rustamov, Alice Wagenaar-Tison, Elysa Doyer, Mathieu Piché

**Affiliations:** 1 Department of Anatomy, Université du Québec à Trois-Rivières, 3351 Boul. Des Forges, C.P. 500, Trois-Rivières, QC G9A 5H7 Canada; 2 CogNAC Research Group, Université du Québec à Trois-Rivières, 3351 Boul. Des Forges, C.P. 500, Trois-Rivières, QC G9A 5H7 Canada

**Keywords:** Irritable bowel syndrome, Selective attention, CPM, DNIC, Electroencephalography, Pain

## Abstract

Irritable bowel syndrome (IBS) is a functional gastrointestinal disorder associated with chronic abdominal pain and altered pain processing. The aim of this study was to examine whether attentional processes contribute to altered pain inhibition processes in patients with IBS. Nine female patients with IBS and nine age-/sex-matched controls were included in a pain inhibition paradigm using counter-stimulation and distraction with electroencephalography. Patients with IBS showed no inhibition of pain-related brain activity by heterotopic noxious counter-stimulation (HNCS) or selective attention. In the control group, HNCS and selective attention decreased the N100, P260 and high-gamma oscillation power. In addition, pain-related high-gamma power in sensorimotor, anterior cingulate and left dorsolateral prefrontal cortex was decreased by HNCS and selective attention in the control group, but not in patients with IBS. These results indicate that the central pain inhibition deficit in IBS reflects interactions between several brain processes related to pain and attention.

## Background

Irritable bowel syndrome (IBS) is a functional gastrointestinal (GI) disorder characterized by chronic abdominal pain and bowel dysfunction [1]. IBS may be associated with psychological distress, impaired quality of life, and disability, and it constitutes a serious societal and economic burden due to its high prevalence [2]. Although several mechanisms have been proposed, the underlying physiopathology of chronic pain in IBS remains unclear.

A consistent finding reported in several studies in patients with IBS is the alteration of central pain inhibition mechanisms [3–10]. Considering that altered pain inhibition is associated with widespread hyperalgesia, it is possible that mechanisms related to diffuse noxious inhibitory controls (DNIC) or conditioned pain modulation (CPM) may be disrupted in IBS. Indeed, DNIC produce diffuse analgesia [11–16] and their dysfunction may cause diffuse pain hypersensitivity. Other factors that may contribute to chronic pain in IBS include altered cognitive functions and attentional processes, which may also lead to widespread pain hypersensitivity independently of the original source of pain.

Regarding cognitive functions, cognitive performance is lower in patients with chronic pain [17] and decreased cognitive inhibition may be associated with reduced pain inhibition [18], although this remains to be demonstrated in patients with chronic pain. In patients with IBS, reduced cognitive performance may affect how pain symptoms are processed and perceived and may contribute to altered pain inhibition, exacerbating chronic pain symptoms. A deficit in episodic visuospatial memory suggests that cognitive functions are altered in patients with IBS [19], although this remains to be replicated [20].

Regarding attention to pain, pain hypervigilance increases pain perception [21, 22]. Also, enhanced attentional capture by pain due to attentional bias towards pain-related information was reported in patients with chronic pain [23, 24]. More specifically, patients with IBS show attentional biases for situational threat words, which is reflected in Stroop facilitation and is associated with gastrointestinal function anxiety [25]. Moreover, they show lower attentional control in an attention network task [26], which could increase attentional capture by pain. Considering that attention to pain generally increases pain perception and that pain inhibition mechanisms such as DNIC/CPM may be modulated by attention [27, 28], enhanced attentional capture by pain could contribute to chronic pain symptoms in IBS.

The aim of this study was to examine whether the modulation of pain-related brain activity by selective attention may be impaired and thus contribute to altered inhibition by heterotopic noxious counter-stimulation (HNCS) in patients with IBS. We also examined whether pain vigilance is increased and whether cognitive inhibition is decreased in patients IBS. We hypothesized that patients with IBS would show decreased inhibition of pain-related brain activity by selective attention and HNCS. Furthermore, we hypothesized that pain hypervigilance and decreased cognitive inhibition would contribute to these alterations in patients with IBS.

## Methods

### Ethics approval

All experimental procedures conformed to the standards set by the latest revision of the Declaration of Helsinki and were approved by the Research Ethics Board of the Université du Québec à Trois-Rivières. All participants gave a written informed consent acknowledging their right to withdraw from the experiment without prejudice and received compensation for their time and commitment to the study. The study consisted of 3 sessions of 90 min each, including the determination of thresholds (pain and the nociceptive flexion reflex NFR) and the evaluation of pain perception, NFR, and somatosensory evoked potentials (SEP).

### Study participants

Patients with IBS: Nine patients with diarrhea-predominant IBS (9 women; mean age ± standard deviation [SD]: 28.9 ± 10.3 years) were recruited for the study by referrals from the gastroenterology unit of the Centre hospitalier affilié universitaire régional de Trois-Rivières (QC, Canada). All patients were evaluated by gastroenterologists experienced in the evaluation of IBS. To make the diagnosis of IBS, normal physical examination, normal colonoscopy with biopsy, exclusion of organic diseases, and the presence of IBS symptoms based on Rome III criteria were required. Patients were excluded if they presented with other chronic pain syndromes, diagnosed psychiatric disorders, or used any medication that could alter pain perception (e.g., analgesics, anxiolytics, antidepressants, and other psychotropic agents) 2 weeks prior to the experiment.

Control group: Nine age- and gender-matched healthy controls (9 women; mean age ± standard deviation [SD]: 28.5 ± 8.5 years) volunteered to participate in the study. They were recruited by advertisements at the campus of the Université du Québec à Trois-Rivières. Participants were included if they had normal bowel habits and no known gastrointestinal disease and were excluded for: (1) taking any medication altering pain perception within 2 weeks prior to the experiment, and (2) having a history of gastrointestinal symptoms, chronic pain, acute or chronic illness, or a diagnosed psychiatric disorder.

### Psychometric assessment

All participants completed the following validated questionnaires before the experiment: the MOS 36-Item Short-Form Health Survey for psychological distress and physical health [29], the Pain Catastrophizing Scale [30], the Pain Vigilance and Awareness questionnaire [31], the Beck Depression Inventory-II [32], and the French version of the State–Trait Anxiety Inventory [33]. In addition, the St-Luc Gastrointestinal Index (GI) was used to assess severity of GI symptoms in patients with IBS [34, 35].

### Assessment of cognitive inhibition

Prior to the experiment, subjects performed a computerized modified Stroop task involving four different conditions (reading, naming, inhibition, and switching), as used previously [36]. In the reading condition, participants read words denoting colors that were displayed in fonts of the same color (red, blue, yellow, or green). In the color-naming condition, participants named the colors of noncolor words that were displayed in one of the same four font colors (neutral condition). In the inhibition condition, participants named the color of the font of incongruent color words (incongruent condition, e.g., the word RED displayed in green font). In the switching condition, participants named the color of the font in which an incongruent color word was displayed when the word is preceded by a cross (similar to the inhibition condition) but read the word when it was preceded by a rectangle. Participants were instructed to perform the tasks as fast and as accurately as possible. In the switching condition, a maximum of 3 naming or reading trials were presented consecutively and performance was decomposed to distinguish the initial switching trial reflecting the change in task-set from the consecutive trials. The analysis of the Stroop interference focused on the consecutive trials following the first switching trial. Performance in these consecutive trials reflected the inhibition process within the context of switching, without including the switching effect. The reaction times (RT) were extracted and the mean value of the 60 trials for each condition was calculated after removing the first four trials to exclude adaptation effects. To calculate the interference effect in the switching condition, the mean RT of the naming condition, serving as a control, was subtracted from the mean RT of the inhibition trials of the switching condition. This calculated interference effect reflects the cost of cognitive inhibition (Stroop interference) in the context of task-switching.

### Experimental design

**Fig. 1:**
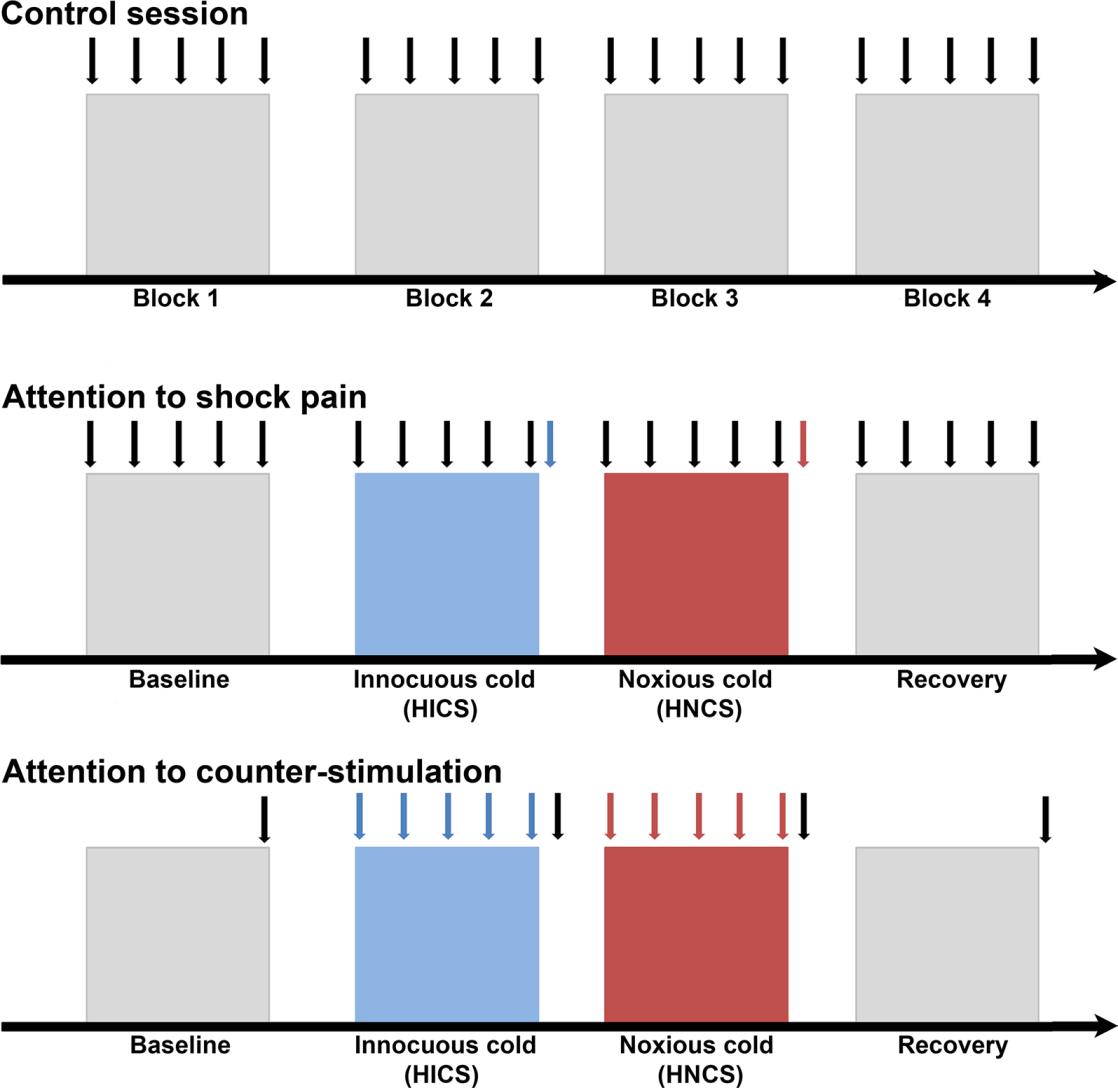
Experimental paradigm. Participants underwent three counterbalanced sessions, including the control session (top panel), attention to shock pain session (middle panel), and attention to counter-stimulation session (bottom panel). Each session included four blocks of 20 painful electrical stimuli delivered at varying intervals of 6–15 s over the right sural nerve. In the attention to shock pain and the attention to counter-stimulation sessions, heterotopic innocuous and noxious counter-stimulation (HICS and HNCS, blue and red blocks, respectively) were applied during the second and third blocks. In the attention to shock pain session, participants were instructed to focus their attention on the shocks and to rate shock pain at regular intervals (black arrows). In addition, they were instructed to rate the coolness of HICS and the pain induced by HNCS at the end of the respective blocks (blue and red arrows, respectively). In the attention to counter-stimulation session, participants were instructed to focus their attention on the counter-stimulation applied on their left forearm (HICS or HNCS). They were instructed to rate HICS or HNCS at regular intervals (blue and red arrows) and shock pain at the end of each block (black arrows)

This study relied on a between-group design to examine the effects of selective attention and HNCS on acute shock pain, the associated electrophysiological responses including SEP, cerebral oscillations and their sources, and the NFR. Each participant completed three 90-min sessions (control session, attention to shock pain session, and attention to counter-stimulation session) separated by a 1–2-week interval (Fig. 1). For the experiment, they sat comfortably on a chair with knee flexion of approximately 120°. In each session, after the NFR threshold assessment, 15 electrical shocks were applied to confirm the NFR stability. For the experimental conditions, 80 painful electrical stimuli were delivered at varying intervals of 6–15 s, distributed in four blocks of 20 stimuli each. In the control session, participants were instructed to pay attention to the electrical shocks. Electrical shocks were applied with no counter-stimulation and participants were prompted to rate shock pain every 5 stimuli and after each block of 20 shocks. In the attention to shock pain and the attention to counter-stimulation sessions, heterotopic innocuous counter-stimulation (HICS) and HNCS were applied during the second and third blocks, respectively. This allowed evaluation of the modulation of brain activity, pain perception, and NFR induced by HNCS and selective attention. Top–down focusing of attention was manipulated across these two sessions. In the attention to shock pain session, participants were instructed to focus their attention on shocks. They were prompted to rate shock pain every 5 stimuli. In addition, they rated the non-painful coolness of HICS and pain induced by HNCS at the end of the respective blocks. In the attention to counter-stimulation session, participants were instructed to focus their attention on the counter-stimulation applied on their left forearm (HICS or HNCS). They were prompted to rate the non-painful coolness of HICS and pain induced by HNCS every 5 stimuli. Additionally, they rated shock pain at the end of each block. Session order was counterbalanced across subjects.

### Painful electrical stimulation (test stimulus)

Transcutaneous electrical stimulation (trains of 10 × 1 ms pulses at 333 Hz) was delivered with an isolated DS7A constant current stimulator (Digitimer Ltd., Welwyn Garden City, Hertfordshire, UK) triggered by a Grass S88 train generator (Grass Medical Instruments, Quincy, MA, USA) and controlled by a computer with a stimulus presentation program (E-Prime2, Psychology Software Tools, Sharpsburg, PA, USA). Degreased skin over the retromalleolar path of the right sural nerve was stimulated by a pair of custom-made surface electrodes (1 cm^2^; 2 cm inter-electrode distance). The NFR threshold was determined using the staircase method including four series of stimuli of increasing and decreasing intensity [8, 37, 38]. Each series always began with a stimulus intensity of 1 mA, followed by stimuli increasing in intensity in increments of 1 mA, reaching a suprathreshold level between 10 and 25 mA (clearly above threshold, but adjusted individually to avoid severe pain). Stimulus intensity was then decreased by steps of 1 mA. After four of these series were completed, the NFR amplitude was plotted against the stimulus intensity (recruitment curve) and threshold was defined as the intensity producing a clear response in at least 50% of trials (responses clearly above background electromyogram [EMG], as defined by the maximum artefact-free EMG activity observed in the same post-stimulus interval [90–180 ms] across all subthreshold stimuli). The pain threshold was defined as the lowest stimulus intensity evoking pain. The intensity of stimulation was then adjusted individually at 120% of the NFR threshold and remained constant during the rest of the experiment.

### Heterotopic noxious counter-stimulation (HNCS: conditioning stimulus)

HNCS was produced by the application of a cold pack on the left forearm (contralateral to the acute shock pain stimulus) for 3 min. This flexible bag (15 × 20 cm), filled with 500 mL of gel, covered most of the anterior forearm surface. When applied, the cold pack was approximately − 12 °C and produced moderate pain.

### Heterotopic innocuous counter-stimulation (HICS: conditioning stimulus)

HICS was delivered with a 9 cm^2^ contact thermode (model TSA-2001; Medoc Advanced Medical Systems, Ramat Yishai, Israel) applied on the anterior surface of the left forearm. Stimulation was continuous for 3 min with slight temperature changes every 10 s (16–17–18–19 °C in pseudo-random order) to reduce the risk of sensory adaptation without introducing major shifts in sensory input. As innocuous stimuli do not typically trigger noxious inhibitory controls [12], HICS was used to control for potential non-specific effects of counter-stimulation. Moreover, HICS was used to direct attention away from the shock pain stimuli by asking subjects to rate the sensation of HICS coolness. Stimulus rating as a distraction task was applied, because it involves cognitive–evaluative processes that are more comparable to those presumably involved in the HNCS paradigm. In typical HNCS paradigms, conditioning stimuli are applied heterotopically, thus activating processes related to spatial attention towards a competing sensory signal.

### Pain ratings

A visual analogue scale (VAS) was shown to participants on a computer monitor to prompt the evaluation of pain produced by electrical stimulation and HNCS. The VAS was arranged horizontally and included the verbal anchors “no pain” and “worst pain imaginable” at the left and right extremities, respectively [39]. HICS was rated on a scale that included the verbal anchors “no sensation” and “cold pain threshold” at the left and right extremities, respectively. Participants were instructed to rate their pain or cold sensation according to these scales, when prompted, by verbally providing numbers between 0 and 100, which corresponded to the left and right anchors of the scales.

### Electroencephalographic recording

Continuous electroencephalogram (EEG) was recorded by means of a 64-channel BrainAmp amplifier, using active Ag–AgCl electrodes that were mounted on an actiCAP in an International 10–20 System montage (Brain Products, Gilching, Germany). Electrode impedance was kept below 10 kΩ. Electrooculographic activity was recorded using a pair of electrodes placed at the suborbital ridge (vertical electrooculogram, vEOG) and at the external ocular canthus (horizontal electrooculogram, hEOG) of the right eye. All EEG electrodes were referenced to the nose with a ground electrode placed on the forehead. EEG and EOG signals were filtered with a 0.01–100 Hz band pass and sampled at 500 Hz for offline analyses.

#### Somatosensory evoked potentials

EEG data was analyzed in MATLAB (Mathworks, Nattick, MA, USA) using EEGLAB version 14_1_1b [40]. Data was filtered offline using a FIR band pass filter with the lower edge at 0.1 Hz and the higher edge at 30 Hz. SEPs were time-locked to the right sural nerve stimulation and baseline-corrected between − 100 ms and 0 prior to the right sural nerve stimulation. Data was screened for extreme values, as well as for infrequent and unstereotyped artifacts using the inbuilt probability function (pop_jointprob) with a threshold of 3 SD56. For further artifact attenuation, Infomax independent component analysis (ICA) was applied. Artifacts were identified using the EEGLAB-Runica function, and independent components (IC) found to reflect blinks, lateral eye movements, muscle-related, and cardiac artifacts were removed from the data. Following ICA-based artifact attenuation, SEPs were averaged for each condition. The amplitude of the P45, N100, and P260 components was quantified using the mean amplitude between fixed latencies (P45: 45–55 ms post-stimulus; N100: 90–120 ms post-stimulus; P260: 280–350 ms post-stimulus).

#### Time–frequency analysis

Time–frequency analysis was performed in the MATLAB environment using EEGLAB version 14_1_1b [40] to examine event-related spectral perturbations (ERSPs). The analysis allows examining brain oscillations that are produced by painful stimuli and how they are modulated by the experimental conditions. It provides further investigation of pain perception and pain regulation mechanisms.

EEG was filtered offline using a FIR band pass filter (1–100 Hz). As described above for ERPs, data was screened for extreme values as well as for infrequent and unstereotyped artifacts, and ICA analysis was applied to remove remaining artifacts. Data was segmented into stimulus-locked epochs from − 1600 to 2600 ms, with time 0 corresponding to the onset of electrical shocks. A Morlet wavelet convolution [41] was computed using the channel time–frequency options available on EEGLAB 14_1_1b [40]. Two hundred time points were generated and 100 linearly spaced frequencies were computed from 1 to 100 Hz. Variable cycles were used for low and high frequencies, with 3 cycles for lowest frequencies and up to 15 cycles for highest frequencies. This allowed the wavelet convolution method to provide a better frequency resolution at lower frequencies and a better temporal resolution at higher frequencies [42]. ERSPs were computed in decibels relative to the − 400 to − 100 ms baseline for each time and frequency point [43, 44].

To examine specific ERSPs, a hypothesis-driven approach was used based on previous studies [67]. Mean power values in four time–frequency maps from the Cz electrode signal were extracted in predetermined regions of interest (time × frequency) from 4 to 10 Hz between 50 and 350 ms, from 8 to 29 Hz between 300 and 1000 ms, from 30 to 60 Hz between 50 and 300 ms, and from 61 to 100 Hz between 100 and 300 ms. The gamma band was separated into low and high frequency components [45]. The ERSP value for each time–frequency point (ERSPtf) included in the regions of interest was calculated for each subject. A mean ERSPtf value was then obtained for each subject over the regions of interest by averaging the values for the 20% highest power (for power increase relative to the baseline) or 20% lowest power (for power decreases relative to the baseline). This procedure has been used in previous studies for time–frequency analysis [46–49] and has the main advantage of allowing the selection of wide regions of interests, thus taking into account variability across subjects while reducing the regression to the mean problem with near-zero values. For each participant, time–frequency data was averaged across all trials per block. The grand average time–frequency maps for the group were obtained by averaging data across subjects for each block.

#### Source estimation analysis

A source estimation package implemented in Brainstorm software was used to estimate the cortical sources of high-gamma oscillations [50]. The forward model was calculated using the Open-MEEG Boundary Element Method [51] on the cortical surface of a template (MNI brain-colin27 atlas) with 1 mm resolution. A noise covariance matrix was estimated from the preprocessed EEG data. Cortical source activation was calculated with a constrained inverse model of EEG sources using the weighted minimum norm current estimation [52] and mapped to a distributed source model consisting of 15,002 elementary current dipoles. We then computed time–frequency decomposition on the source time series for each trial using the Morlet transform from 60 to 100 Hz in 1 Hz steps. The resulting maps across trials were averaged for each subject. Consistent with the time–frequency analysis on the electrodes, the source analysis focused on the time window of the high-gamma oscillations (100–300 ms). Two-tailed *t* tests between conditions (attention to shock pain session, HNCS vs. baseline; attention to counter-stimulation session, HICS vs. baseline) were applied to each point in space to identify statistically significant voxels (frequency range 70–90 Hz, time window 0–300 ms). To minimize the possibility of erroneous results, we presented source estimations if the statistically significant differences at the source level survived the 5% FDR-based multiple comparison correction.

### NFR measurement and analysis

Electromyography (EMG) of the short head of the right biceps femoris was recorded with a pair of surface electrodes (EL-508, Biopac Systems, Inc., Goleta, CA, USA). It was amplified 1000 times, band pass filtered (10–500 Hz), sampled at 1000 Hz (Biopac Systems), and stored on a personal computer for offline analysis using Acknowledge 4.1.1 software. The raw EMG recording was full-wave rectified and the resulting signal was used to quantify the amplitude of the NFR to each shock by extracting the integral value between 90 and 180 ms after the stimulus onset [8, 18, 27, 28, 37, 53, 54]. This amplitude was normalized for each electrical stimulus using a t-transformation. The mean of 20 responses in each block was calculated to compare blocks within and between sessions.

### Statistical analyses

All results are expressed as mean ± SD unless specified. The data was analyzed with Statistica v13.1 (Dell Inc. 2016, Tulsa, OK, USA) with significance thresholds set to *p* ≤ 0.05. Group differences in psychological variables were assessed by *t* tests for independent samples. The modulation of SEPs, ERSPs, pain ratings, and NFR between groups across sessions and blocks was assessed by mixed analysis of variance (ANOVA) with Group (patients with IBS, control group) as the between subject factor and Session (control, attention to shock pain, and attention to counter-stimulation) and Block (baseline, HICS, HNCS, and recovery) as within-subject factors. Planned contrasts were used to decompose significant effects and to test a priori hypotheses for inhibitory effects of HNCS and selective attention. Effect sizes are reported based on partial eta-squared ($\eta_{p}^{2}$).

## Results

### Groups characteristics

Characteristics of patients with IBS and the control group are reported in Table 1. The mean duration of IBS symptoms was 11.0 ± 2.5 years and the severity of gastrointestinal symptoms was moderate (St-Luc scale: 69.4 ± 19.3). Patients with IBS showed significantly lower mental health (45 ± 8.8 vs. 56.1 ± 6.7, *p* < 0.008) and physical health (43 ± 5.5 vs. 52.3 ± 4.8, *p* < 0.001) compared with the control group. Moreover, patients with IBS reported higher pain vigilance (Pain Awareness and Vigilance Questionnaire: 43.3 ± 8.8 vs. 27.8 ± 14.3, *p* < 0.01) and pain catastrophizing (Pain Catastrophizing Questionnaire: 24.1 ± 12 vs. 10.9 ± 8.5, *p* < 0.02) compared with the control group. The NFR threshold was significantly lower in patients with IBS compared with the control group (10.7 ± 4.8 vs. 15.4 ± 3.5, *p* < 0.02), but baseline shock pain ratings were not significantly different between groups (46.3 ± 15.9 vs. 41.6 ± 17.2, *p* = 0.3). In the Stroop test, reaction times (RT) in the inhibition trials of the switching condition were higher in patients with IBS compared with the control group although this effect did not reach statistical significance (982 ± 170 ms vs. 842 ± 109 ms, *p* = 0.054), while RT were not significantly different in the naming condition (754 ± 36 ms vs. 663 ± 36 ms, *p* = 0.10).

**Table 1:** Characteristics of participants (mean ± SD)

	Age (years)	GI symptoms severity	Duration (years)	Mental health	Physical health	PC	Vigilance	Depression	State anxiety	Trait anxiety
Patients with IBS (*n* = 9)	28.9 ± 10.3	69.4 ± 19.3	11 ± 2.5	45 ± 8.8^†^	43 ± 5.5^†^	24.1 ± 12*	43.3 ± 8.8*	8.3 ± 7.30	34.8 ± 9.70	41.8±9.60
Healthy controls (*n* = 9)	28.5 ± 8.5	n.a.	n.a.	56.1 ± 6.7	52.3 ± 4.8	10.9 ± 8.5	27.8 ± 14.3	3.9 ± 7	28 ± 8.5	33.8±11.4

n.a.: not applicable; GI: gastrointestinal; PC: pain catastrophizing; *p < 0.05; †*p* < 0.01

### Altered inhibition of pain-related brain activity by heterotopic noxious counter-stimulation (HNCS) and selective attention in patients with IBS

**Fig. 2:**
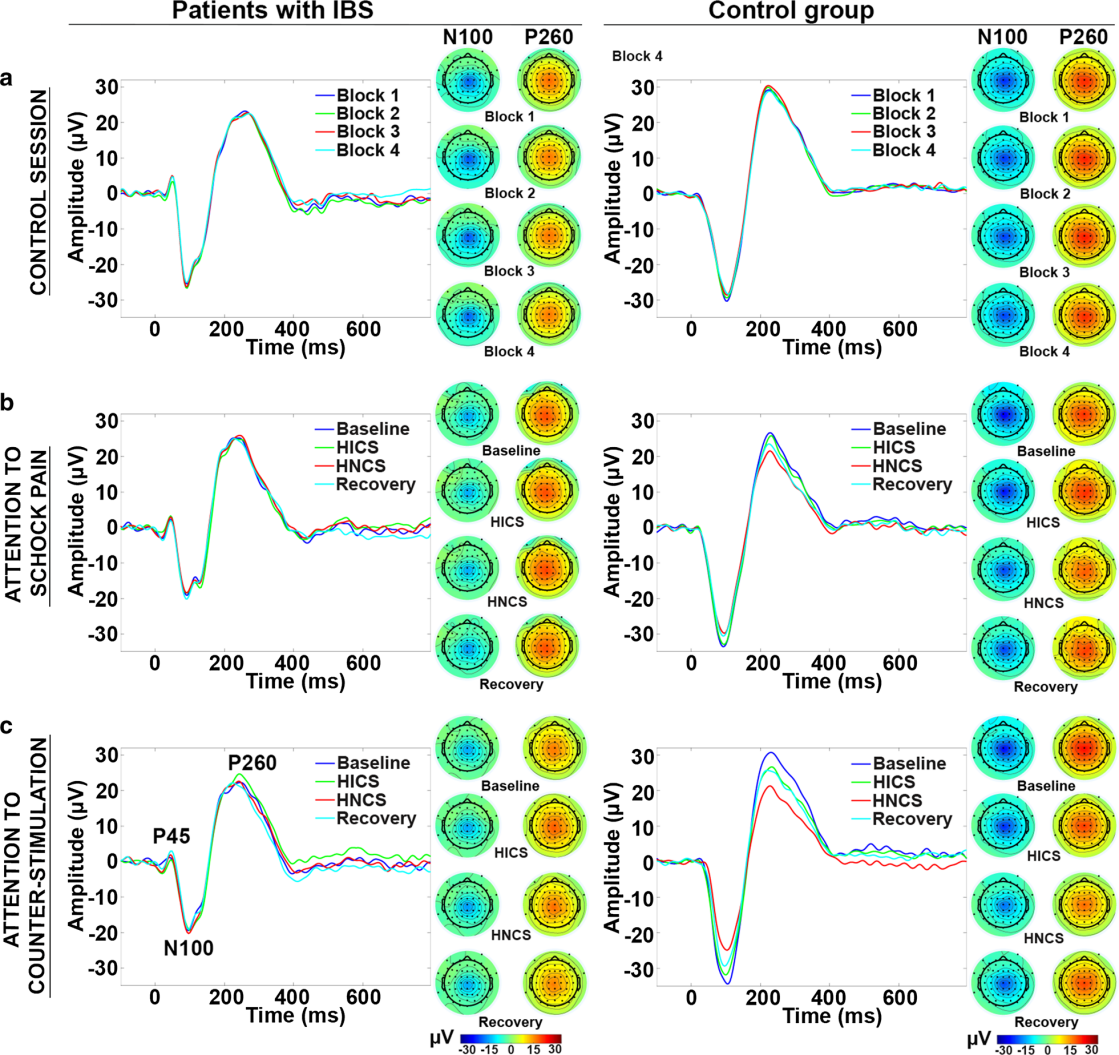
Modulation of somatosensory evoked brain potentials by selective attention and heterotopic noxious counter-stimulation (HNCS). Grand average somatosensory evoked potentials (SEP) recorded at Cz and scalp topography of components showing significant modulation by selective attention and HNCS (N100 and P260) are presented for each block, session, and group (patients with IBS, left column; control group, right column). The three components that were analyzed (P45, N100, and P260) are indicated on the bottom left panel. For mean amplitudes of the components and statistical results, see Table 2, Fig. 3, and the results section. *X*-axis, time in ms; *Y*-axis, amplitude in μV. IBS: irritable bowel syndrome; HICS: heterotopic innocuous counter-stimulation; HNCS: heterotopic noxious counter-stimulation

**Fig. 3:**
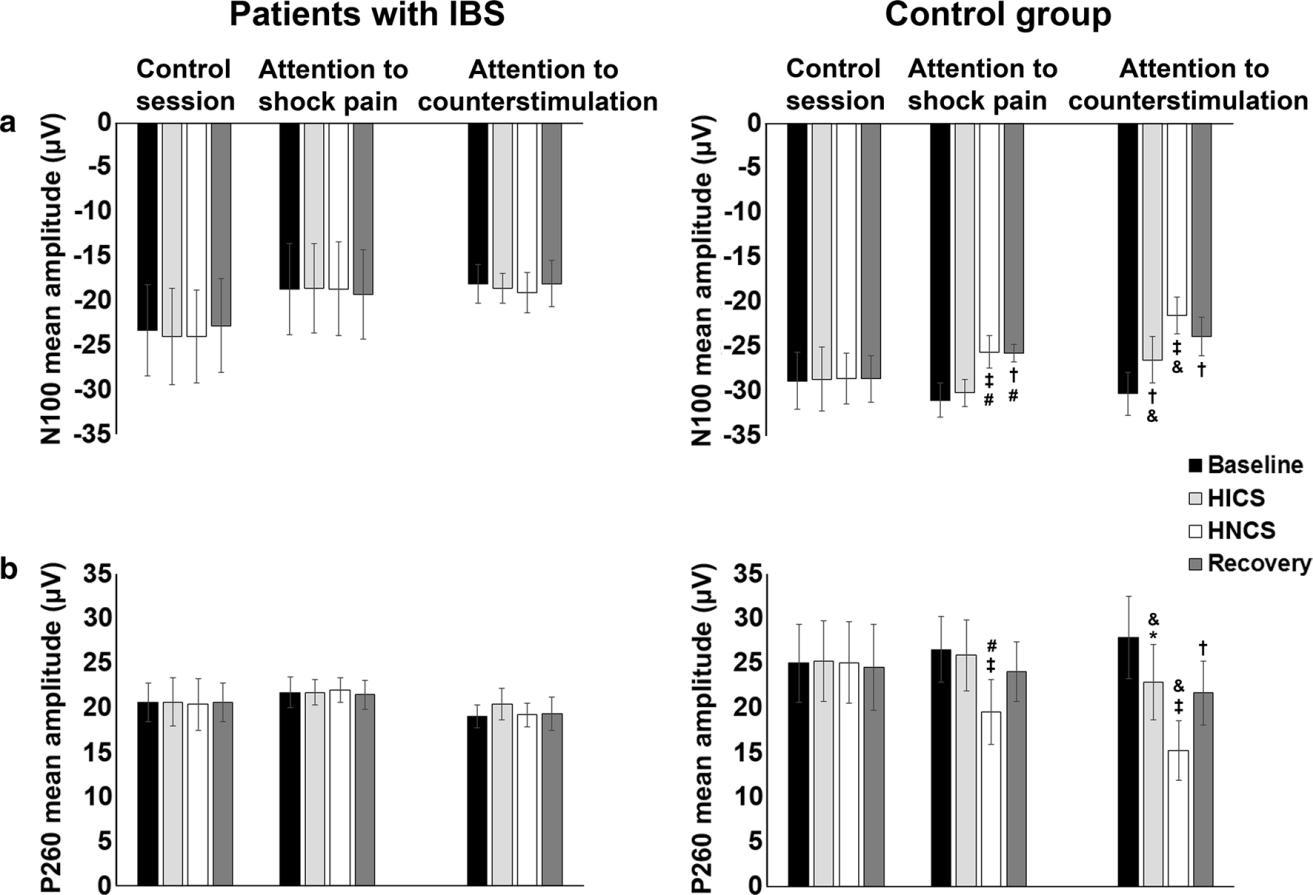
Modulation of somatosensory evoked brain activity by selective attention and heterotopic noxious counter-stimulation (HNCS). Amplitude (mean ± SEM) of the N100 and P260 components (**a**, **b**, respectively) for each block, session, and group (patients with IBS, left column; control group, right column). For mean amplitudes of the components and statistical results, see Table 2 and “Results” section. The inhibitory effect of HICS and HNCS compared with baseline within each session is represented by the *, ^†,^ and ^‡^ symbols. *p < 0.05, ^†^*p *< 0.01, ^‡^*p *< 0.001. The inhibitory effect of HNCS compared with HICS vs. the corresponding changes in the control session is represented by the # symbol. The inhibitory effect of HICS compared with baseline and HNCS compared with HICS vs. the corresponding changes in the attention to shock pain session are represented by the & symbol. ^#^*p* < 0.001; ^&^*p* < 0.05. *X*-axis, conditions and sessions; *Y*-axis, amplitude in μV. NRS: numerical rating scale; SEM: standard error of the mean; IBS: irritable bowel syndrome; HICS: heterotopic innocuous counter-stimulation; HNCS: heterotopic noxious counter-stimulation

The grand averages of SEPs and scalp topography of the N100 and P260 components are presented in Fig. 2. The comparison of mean amplitudes between groups across sessions and blocks for the P45, N100, and P260 measured at Cz is reported in Table 2 and Fig. 3. Statistical within-session comparisons are also presented in Table 2 and Fig. 3.

**Table 2:** Effects of HNCS and selective attention on pain and pain responses (mean ± SD)

	Control session				Attention to shock pain				Attention to counter-stimulation			
	Baseline	HICS	HNCS	Recovery	Baseline	HICS	HNCS	Recovery	Baseline	HICS	HNCS	Recovery
Patients with IBS (*n* = 9)												
P45 (µV)	4.7 ± 3.8	4.4 ± 3.3	4.8 ± 2.4	4.7 ± 2.9	4 ± 2.7	4.1 ± 3.2	4.0±3.6	3.5 ± 2.9	1.9 ± 1.7	2.2 ± 1.3	2.1 ± 2.0	2.5 ± 2.1
N100 (µV)	− 23.3 ± 14	− 24.0 ± 15.3	− 24.0 ± 14.9	− 22.8 ± 14	− 18.7 ± 14	− 18.6 ± 14.0	− 18.6 ± 14.9	− 19.3 ± 14.2	− 18 ± 6.1	− 18.6 ± 4.7	− 19.1 ± 6.4	− 18 ± 7.3
P260 (µV)	20.6 ± 6.2	20.6 ± 7.6	20.3 ± 8.2	20.6 ± 6.1	21.7 ± 4.9	21.7 ± 4.0	22 ± 3.9	21.4 ± 4.6	19 ± 3.7	20.4 ± 5.1	19.2 ± 3.8	19.3 ± 5.4
4–10 Hz	12.3 ± 3.7	12.0 ± 2.9	12.0 ± 2.8	11.2 ± 2.9	12 ± 2.1	11.6 ± 3.1	12.1 ± 3.8	11.1 ± 2.4	12.4 ± 2.4	11.4 ± 1.9	11.5 ± 3.2	13.4 ± 3.4
8–29 Hz (ERS)	3.9 ± 2.8	2.4 ± 1.1	3.1 ± 2.2	3.7 ± 2.7	3.2 ± 1.9	2.6 ± 2.0	2.9 ± 2.4	2.7 ± 1.9	3.3 ± 2.9	2.9 ± 2.6	2.7 ± 1.6	3,8 ± 2.7
8–29 Hz (ERD)	− 3.0 ± 1.1	− 2.1 ± 0.9	− 2 ± 0.7	− 1.9 ± 1.3	− 1.7 ± 1.2	− 2.6 ± 1.6	− 1.6 ± 1.3	− 2.3 ± 1	− 3,2 ± 1.5	− 2,3 ± 1.3	− 2,9 ± 1.0	− 1.6 ± 1.1
30–60 Hz	3.0 ± 1.1	3.0 ± 0.8	3.8 ± 2.8	3.0 ± 1.3	2.7 ± 1.2	2.6 ± 1.7	3.6 ± 1.9	2.9 ± 1.4	2.5 ± 1.3	2.5 ± 1.3	3.3 ± 1.1	3.5 ± 1.6
61–100 Hz	3.3 ± 0.8	3.3 ± 1.6	2.9 ± 0.8	3.1 ± 1.7	3.0 ± 1.2	3.2 ± 1.5	3.5 ± 1.6	3.2 ± 1.6	3.0 ± 1	3.3 ± 2	3.5 ± 1.4	3.0 ± 1.7
Shock pain (NRS)	45.6 ± 18.2	51.3 ± 22.6	50.8 ± 24	48.8 ± 25.2	49.6 ± 17.5	49.9 ± 18.2	46.8 ± 15.8	48.8 ± 15.5	43.9 ± 15	41.4 ± 14.2	41.3 ± 9	42.9 ± ± 15
NFR (T− score)	50.3 ± 6.2	49.4 ± 2	50.3 ± 2.6	50.1 ± 4.5	51.9 ± 3.8	49.8 ± 2.6	51.3 ± 3.7	50.2 ± 4.1	52.1 ± 4.5	50.2 ± 2.9	51.9 ± 6.1	49.7 ± 5.3
Healthy controls (*n* = 9)												
P45 (µV)	− 0.6 ± 3.8	− 0.6 ± 4.0	− 0.2 ± 4.2	− 0.7 ± 4.6	− 0.3 ± 1.9	− 0.7 ± 2.6	− 0.9 ± 2.5	0.1 ± 3.2	− 0.2 ± 2.5	− 0.5 ± 3	− 0.3 ± 2.9	0.5 ± 2.1
N100 (µV)	− 28.8 ± 9.6	− 28.6 ± 10	− 28.6 ± 8	− 28.6 ± 7.3	− 30.9 ± 5.40	− 30.1 ± 4.40	− 25.5 ± 5.2^‡^	− 25.7 ± 2.7^†^	− 30.3 ± 6.7	− 26.5 ± 7.3^†^	− 21.5 ± 5.7^‡^	− 23.8 ± 6.1^†^
P260 (µV)	25 ± 13.2	25.2 ± 13.5	25.0 ± 13.6	24.5 ± 13.6	26.5±10.4	25.9 ± 11.2	19.5 ± 10.2^‡^	24 ± 9.4	27.8 ± 13.1	22.8±11.9*	15.2 ± 9.4^‡^	21.6 ± 10.2^†^
4–10 Hz	11.5 ± 2.3	12.6 ± 2.1	12.2 ± 3.0	11.0±1.8	13.3 ± 2.5	12.7±2	11.3 ± 3.2	11.7 ± 2.8	12.7 ± 2	11.5 ± 2.8	11.2±2.6	11.7 ± 3.2
8–29 Hz (ERS)	2.7 ± 1.1	3.0±1.6	2.4 ± 1.0	3.8 ± 2.3	2.9 ± 1.5	3.2 ± 1.6	3.0 ± 1.7	3.4±2.1	2.0 ± 1.5	2.6 ± 1.6	2.4 ± 1.2	2.2±1.2
8–29 Hz (ERD)	− 2.3 ± 1.1	− 1.9 ± 0.8	− 2.2 ± 0.8	− 1,8 ± 0.7	− 1.8 ± 0.8	− 1,7 ± 0.4	− 1.9 ± 0.8	− 2.3 ± 1.3	− 1,9 ± 1.1	− 2.0 ± 0.6	− 1.7 ± 0.8	− 2.0 ± 0.6
30–60 Hz	1.9 ± 0.8	2.0 ± 0.4	2.1 ± 0.8	2.1 ± 0.6	2.5 ± 1.1	2.2 ± 0.7	1.7 ± 0.6*	1.6 ± 0.7*	2.3 ± 0.8	1.9 ± 0.9^†^	1.5 ± 0.6	1.9 ± 0.8
61–100 Hz	3.1 ± 2.0	2.9 ± 1.4	3.3 ± 1.2	3.1 ± 0.9	3.5 ± 1.3	3.0 ± 1.5	1.3 ± 0.6^†^	2.1 ± 0.6*	3.8 ± 1.6	2.1 ± 0.5*	1.5 ± 0.4^†^	2.2 ± 0.6*
Shock pain (NRS)	37.8 ± 15.1	38.9 ± 14.3	41.8 ± 17.0	41.7 ± 16.1	43.6 ± 18.3	42.6 ± 18.6	38.2 ± 20.2*	42.7 ± 18.3	43.5 ± 23.2	34.9 ± 21*	28 ± 18.8^†^	41.1 ± 21.1
NFR (T− score)	49.7 ± 3.4	50.5 ± 2.8	50.8 ± 3.2	48.9 ± 2.9	51.1 ± 3.2	49.1 ± 3.8	51.6 ± 4.2	49.2 ± 3.5	50.5 ± 4.1	50.5 ± 4.4	53.5 ± 4.7	47.2 ± 3.8

*p < 0.05; ^†^*p* < 0.01; ^‡^*p* < 0.001

#### P45 mean amplitude

The P45 mean amplitude was not significantly different between groups across sessions and blocks (interaction: F_6,96_ = 0.4, *p* = 0.9, $\eta_{p}^{2}$ = 0.02). Also, it was not significantly different between sessions across blocks (interaction: F_6,96_ = 0.3, *p* = 0.9, $\eta_{p}^{2}$ = 0.02). These results indicate that HNCS and selective attention did not modulate the P45 significantly.

#### N100 and P260 mean amplitude

The N100 and P260 mean amplitudes were significantly different between groups across sessions and blocks (interaction: F_6,96_ = 4.8, *p* < 0.001, $\eta_{p}^{2}$ = 0.23 and F_6,96_ = 5.7, *p* < 0.001, $\eta_{p}^{2}$ = 0.26, respectively). These interactions were then decomposed using planned contrasts to test *a priori* hypotheses.

In the control session, the mean amplitudes of the N100 and P260 were not significantly different in the second, third, or fourth blocks compared with the baseline block for either patients with IBS (all *p* > 0.4) or controls (all *p* > 0.5). This indicates that non-specific temporal changes in N100 and P260 were not significant.

In the attention to shock pain session, HICS did not significantly modulate the N100 and P260 in comparison to baseline in either patients with IBS (both *p* > 0.5) or controls (both *p* > 0.3), indicating a lack of N100 and P260 modulation by heterotopic non-painful cold stimulation. In contrast, HNCS decreased the N100 and P260 compared with HICS and these effects were significant compared with the corresponding changes in the control session, for controls compared with patients with IBS (both *p* < 0.01). Indeed, N100 and P260 inhibition by HNCS was significant in controls (both *p* < 0.001) but not in patients with IBS (both *p* > 0.6). This indicates that HNCS inhibited the N100 and P260 after accounting for non-specific temporal changes in controls, but not in patients with IBS. In controls, the N100 remained significantly decreased during recovery compared with the HICS block, vs. the corresponding changes in the control session (*p* < 0.001), but this was not the case for the P260 (*p* = 0.4).

In the attention to counter-stimulation session, the mean amplitude of the N100 and P260 was significantly decreased by HICS compared with baseline vs. the corresponding changes when attention was focused on shock pain, and these effects of selective attention were significantly different between groups (both *p* < 0.03). Indeed, selective attention inhibited the N100 and P260 in controls the control group (both *p* < 0.02) but not in patients with IBS (both *p* > 0.4). This indicates that selective attention inhibited the N100 and P260 after accounting for temporal non-specific changes in the control group, but not in patients with IBS. Moreover, HNCS produced significant N100 and P260 inhibition compared with HICS, vs. the corresponding changes in the attention to shock pain session, and these effects were significantly different between groups (both *p* < 0.04). Indeed, these effects were significant in the control group (both *p* < 0.02) but not in patients with IBS (both *p* > 0.4), suggesting that HNCS and selective attention produced additive inhibition of brain activity related to the N100 and P260 in the control group only. This also suggests that the lack of inhibition by selective attention may contribute to altered inhibition by HNCS in IBS, which is supported by a significant correlation between inhibition of the N100 by HNCS and selective attention (*r* = 0.47; *p* = 0.050) and inhibition of the P260 by HNCS and selective attention (*r* = 0.50; *p* = 0.035).

#### Event-related spectral perturbations

**Fig. 4:**
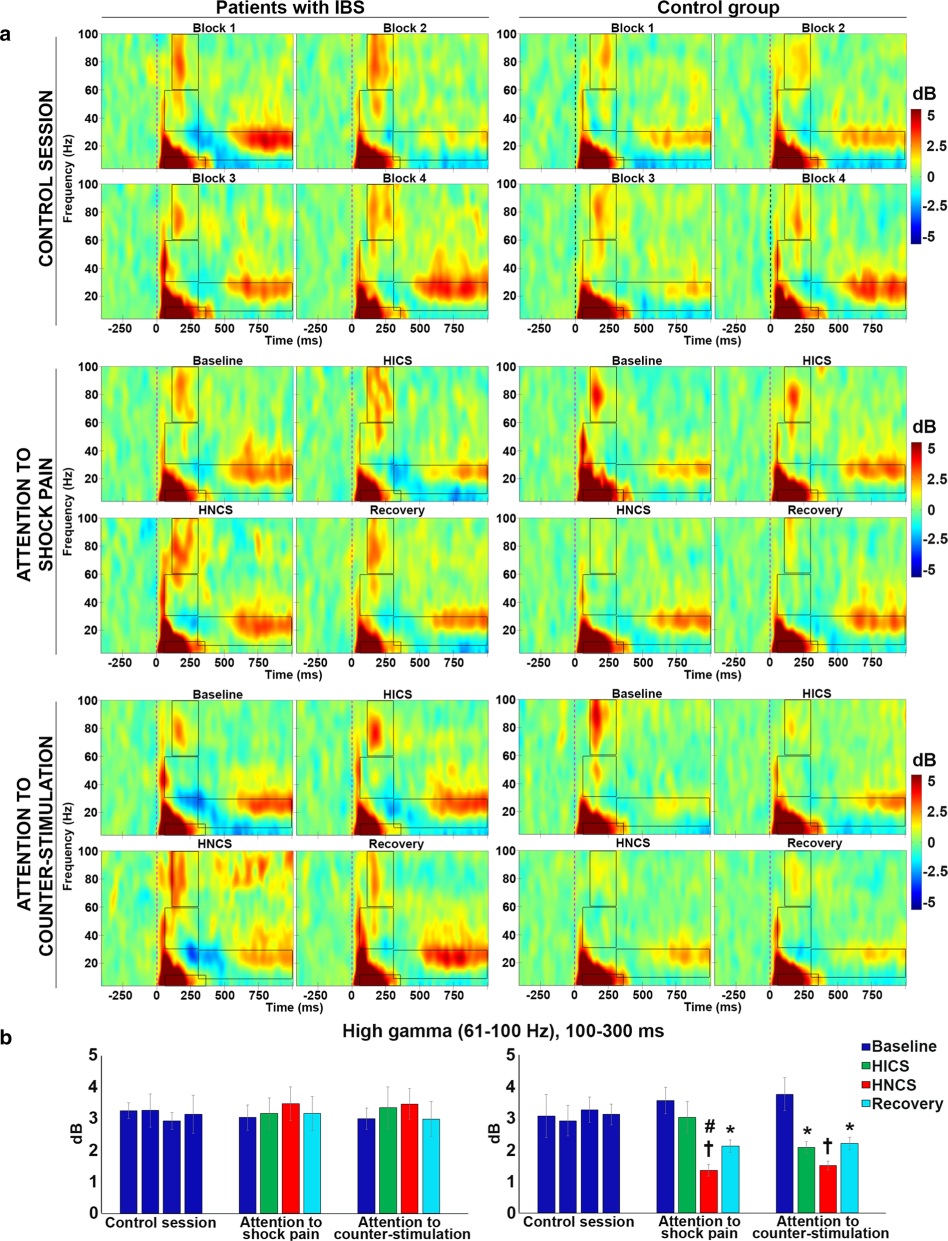
Modulation of event-related spectral perturbations by selective attention and heterotopic noxious counter-stimulation (HNCS). **a** Mean stimulus-locked event-related spectral perturbations at electrode Cz. The dashed line indicates the stimulus onset. Oscillation power is presented in dB relative to a pre-stimulus baseline (−400 ms to −100 ms). Positive and negative power changes are represented by red and blue colors, respectively. Rectangles represent the following predetermined regions of interest (time x frequency): 4–10 Hz, 50–350 ms; 8–29 Hz, 300–1000 ms; 30–60 Hz, 50–300 ms; 61–100 Hz, 100–300 ms. The mean power values for the regions of interest (time × frequency) calculated according to the top 20% approach (see “Methods” section) and statistical results are illustrated in Table 2 and in “Results” section. *X*-axis, time in ms; *Y*-axis, frequency in Hz. **b** Power value (mean ± SEM) in dB for high-gamma oscillations (61–100 Hz, 100–300 ms). The inhibitory effect of HICS and HNCS compared with baseline within each session is represented by the * and ^†^ symbols. *p < 0.05, ^†^*p* < 0.01. The inhibitory effect of HNCS compared with HICS vs. corresponding changes in the control session is represented by the ^#^ symbol. ^#^*p* > 0.05. *X*-axis, conditions and sessions; *Y*-axis, power values in dB. SEM: standard error of the mean; IBS: irritable bowel syndrome; HICS: heterotopic innocuous counter-stimulation; HNCS: heterotopic noxious counter-stimulation

To examine cerebral processes underlying the reduction in brain activity induced by HNCS and selective attention, the modulation of pain-related brain oscillations was investigated using ERSP analyses. ERSP analysis was based on regions of interests in the time–frequency map of Cz (see Fig. 4 and Table 2).

The mean power of high-gamma oscillations was significantly different between groups across sessions and blocks (interaction: F_6,96_ = 2.2, *p* = 0.047, $\eta_{p}^{2}$ = 0.12; see Table 2 and Fig. 4). No effect was observed between groups across sessions and blocks for regions of interest in other frequency bands (see Table 2). Statistical within-session comparisons are also presented in Table 2 and Fig. 4.

Planned contrasts revealed that in the control session, the mean power of high-gamma oscillations was not significantly different in the second, third, and fourth blocks compared with the baseline block for either patients with IBS (all *p* > 0.3) or the control group (all *p* > 0.3). This excludes temporal non-specific changes in the mean power of high-gamma oscillations.

In the attention to shock pain session, HICS did not significantly modulate high-gamma oscillations in comparison to baseline either in patients with IBS (both *p* > 0.3) or the control group (both *p* > 0.2), indicating a lack of high-gamma modulation by heterotopic non-painful cold stimulation. In contrast, HNCS decreased the mean power of high-gamma oscillations compared with HICS, vs. the corresponding changes in the control session, and this effect of HNCS was significantly different between groups (*p* < 0.04). Indeed, HNCS inhibited high-gamma oscillations in the control group (*p* < 0.04) but not in patients with IBS (*p* = 0.4). After HNCS, the mean power of high-gamma oscillations did not remain significantly decreased compared with the HICS block vs. the corresponding changes in the control session (*p* = 0.6). This indicates that pain-related brain processes underlying cerebral oscillations in the high-gamma range are inhibited during the heterotopic application of another painful stimulus in healthy individuals, but not in patients with IBS.

In the attention to counter-stimulation session, the mean power of high-gamma oscillations was not significantly modulated by HICS compared with baseline, vs. the corresponding changes in the attention to shock pain session for the control group compared with patients with IBS (*p* = 0.13). Indeed, no effect of selective attention was observed for either patients with IBS (*p* > 0.2) or the control group (*p* > 0.2). In addition, high-gamma oscillations were not significantly modulated by HNCS compared with HICS, vs. the corresponding contrast in the attention to shock pain session for the control group compared with patients with IBS (*p* > 0.3). Indeed, this effect was not significant either in patients with IBS (*p* > 0.7) or the control group (*p* > 0.3), indicating that HNCS and selective attention did not produce significant additive inhibition of high-gamma oscillations, consistent with the lack of effect of selective attention.

### Source estimation of high-gamma oscillation modulation

**Fig. 5:**
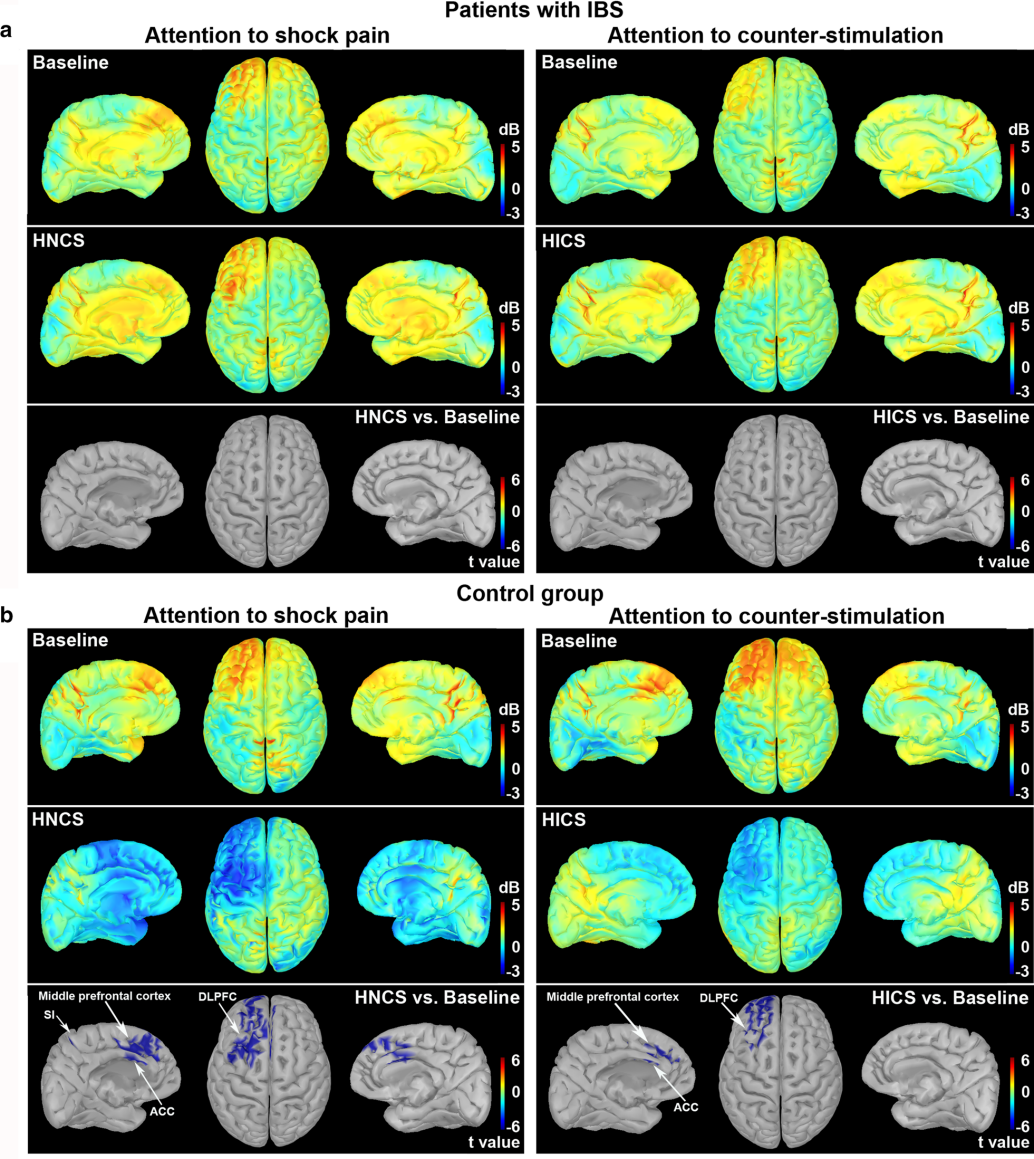
Modulation of pain-related high-gamma oscillation sources by selective attention and heterotopic noxious counter-stimulation (HNCS). Source estimation of high-gamma oscillations (at 86 Hz, 158 ms post-stimulation) represented as t values, based on a voxelwise two-tailed paired *t* test on time–frequency source space (**a** patients with IBS; **b** control group). Time courses of high-gamma oscillations during HNCS and HICS blocks were compared to those during baseline block. Positive and negative relationships are depicted by warm and cool colors, respectively. Whole-brain t-maps were thresholded at *p* < 0.05, false discovery rate corrected for the whole brain. Statistical results and MNI coordinates of strongest relationships (peak locations) are provided in the “Results” section. Hz: hertz; ms: milliseconds; SI: primary somatosensory cortex; ACC: anterior cingulate cortex; DLPFC: dorsolateral prefrontal cortex; IBS: irritable bowel syndrome; HICS: heterotopic innocuous counter-stimulation; HNCS: heterotopic noxious counter-stimulation

To examine the sources of high-gamma power decrease by HNCS and selective attention compared with baseline, source estimation was calculated. At baseline, pain-related activity induced by the shock-evoked robust gamma oscillations in several pain-related areas in both groups (bilateral foot region of primary somatosensory cortex [SI], lateral and medial prefrontal cortex, premotor cortex, cingulomotor area) (Fig. 5a, b, upper and middle rows, right and left panels). Voxelwise two-tailed paired *t* tests on time–frequency source space were then applied: attention to shock pain session, HNCS vs. baseline (effect of HNCS); and attention to counter-stimulation session, HICS vs. baseline (effect of selective attention). In patients with IBS, neither HNCS nor selective attention caused significant changes in cortical foci of high-gamma power (Fig. 5a, bottom row, right and left panels). In the control group, selective attention resulted in statistically significant foci of high-gamma power decreases compared with baseline. The foci were located in the left lateral prefrontal cortex (including left DLPFC, MNI: −46, 38, 8), left medial prefrontal cortex (MNI: −2, 45, 36), and left anterior cingulate cortex (ACC) (MNI: −1, 27, 19) (Fig. 5b, right panels). These effects were observed from 154 to 162 ms post-stimulation, mostly at 70–90 Hz, peaking at 158 ms and 84 Hz (Fig. 5b, bottom row, right panels). Similarly, HNCS significantly decreased high-gamma power compared with baseline. The foci were located in the left lateral prefrontal cortex (including left DLPFC, MNI: −46, 38, 8), left premotor regions (−3, 7, 71), bilateral medial prefrontal cortex (left, −2, 45, 36 and right, 1, 43, 40), bilateral ACC (left, −1, 27, 19 and right, 0, 32, 23), and left foot region of primary somatosensory cortex (SI, −6, −42, 79) (Fig. 5b, bottom row, left panels).

### Modulation of pain perception

**Fig. 6:**
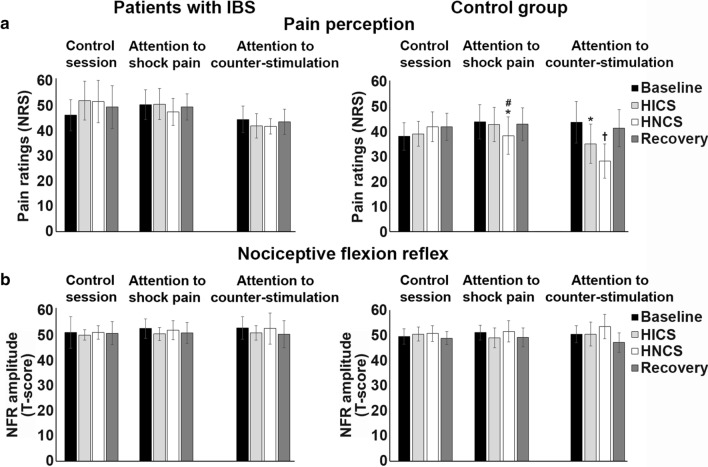
Modulation of pain perception and NFR amplitude by selective attention and heterotopic noxious counter-stimulation (HNCS). **a** Pain ratings (mean ± SEM) of the electrical stimulation for each block, session, and group (patients with IBS, left column; control group, right column). For mean values and statistical tests, see Table 2 and “Results” section. The inhibitory effect of HICS and HNCS compared with baseline within each session is represented by the * and ^†^ symbols. *p < 0.05, ^†^*p* < 0.01. The inhibitory effects of HNCS compared with HICS vs. the corresponding changes in the control session is represented by the ^#^ symbol. ^#^*p* > 0.05. *X*-axis, conditions and sessions; *Y*-axis, pain ratings of the electrical stimulation. **b** Standardized NFR amplitude (T-score ± SEM) induced by the electrical stimulation for each block, session, and group (patients with IBS, left column; control group, right column). For mean values and statistical results, see Table 2 and the “Results” section). *X*-axis, conditions and sessions; *Y*-axis, standardized amplitude (T-score). NRS: numerical rating scale; SEM: standard error of the mean; IBS: irritable bowel syndrome; HICS: heterotopic innocuous counter-stimulation; HNCS: heterotopic noxious counter-stimulation

Shock pain ratings were compared between groups and across experimental sessions and blocks by a mixed ANOVA (see Table 2). Pain ratings were not significantly different between groups across sessions and blocks (interaction: F_6,96_ = 0.8, *p* = 0.6, $\eta_{p}^{2}$ = 0.05). However, pain ratings were significantly different between sessions across blocks (interaction: F_6,96_ = 3.2, *p* < 0.006, = 0.17; see Table 2 and Fig. 6). This interaction was decomposed using planned contrasts to test *a priori* hypotheses for simple effects (group by group). Statistical within-session comparisons are also presented in Table 2 and Fig. 6.

In the control session, pain ratings were not significantly different in the second, third, or fourth blocks compared with the baseline block for either patients with IBS (all *p* > 0.1) or c the control group (all *p* > 0.3), except for a slight increase of pain ratings in the second block compared with baseline block in patients with IBS (*p* < 0.03).

In the attention to shock pain session, HICS did not significantly modulate pain ratings in comparison to baseline in the control group (*p* = 0.4), but patients with IBS showed increased pain perception (*p* = 0.05). In contrast, HNCS significantly decreased pain ratings compared with HICS in comparison to the corresponding changes in the control session in the control group (*p* < 0.05) but not in patients with IBS (*p* > 0.5), indicating that HNCS produced the expected hypoalgesic effect in the control group after accounting for temporal non-specific changes. After HNCS, however, pain ratings did not remain significantly decreased in the control group during recovery compared with HICS, vs. the corresponding changes in the control session (*p* = 0.5).

In the attention to counter-stimulation session, pain ratings were not significantly modulated by HICS compared with baseline, vs. the corresponding changes in the attention to shock pain session in either patients with IBS (*p* = 0.2) or the control group (*p* = 0.1), indicating that selective attention did not significantly decrease pain. Moreover, pain inhibition by HNCS compared with HICS was not significantly different, vs. the corresponding changes in the attention to shock pain session either in patients with IBS (*p* > 0.9) or the control group (*p* > 0.1), suggesting that HNCS and selective attention did not produce significant additive hypoalgesia, consistent with the lack of pain inhibition by selective attention. Additionally, the ability to decrease pain by selective attention may contribute to pain inhibition by HNCS or its alteration in IBS. Accordingly, pain inhibition amplitude by HNCS was significantly associated with pain inhibition amplitude by selective attention (*r *= 0.60; *p *= 0.0008), consistent with the same associations observed for the N100 and P260.

### Nociceptive flexion reflex

NFR amplitude was not significantly different between groups across sessions and blocks (interaction: F_6,96_ = 0.2, *p* = 0.9, $\eta_{p}^{2}$ = 0.01; see Table 2). Also, it was not significantly different between sessions across blocks (interaction: F_6,96_ = 0.6, *p* = 0.7, $\eta_{p}^{2}$ = 0.04; see Table 2 and Fig. 6). These results indicate that HNCS and selective attention did not significantly modulate the NFR in the present experimental conditions.

### Contribution of pain hypervigilance to altered inhibition of pain-related brain activity

To examine whether group differences in pain vigilance may contribute to decreased inhibition of pain-related brain activity by HNCS and selective attention, covariance analyses were conducted.

The effects of HNCS and selective attention on the N100 and P260 reported above were no longer significant after controlling for group differences in pain vigilance (interaction: F_6,84_ = 2.0, *p* = 0.07, $\eta_{p}^{2}$ = 0.13 and F_6,84_ = 1.3, *p* = 0.3, $\eta_{p}^{2}$ = 0.08, respectively), but remained significant for high-gamma power (interaction: F_6,84_ = 2.3, *p* = 0.045, $\eta_{p}^{2}$ = 0.14).

## Discussion

The present results are consistent with and extend those from previous reports indicating that patients with IBS show altered pain inhibition processes. In addition to altered inhibition of pain and pain-related brain activity by HNCS, patients with IBS showed altered inhibition of pain-related brain activity by selective attention. Moreover, high-gamma oscillations in ACC and left DLPFC were decreased by HNCS and selective attention in the control group. The same sources were observed in patients with IBS, but their activity was not significantly affected by HNCS and selective attention. In addition, patients with IBS showed increased pain vigilance, which contributed to some of the above-mentioned alterations. Altogether, these results indicate that decreased pain inhibition by HNCS in patients with IBS reflects interactions between several brain processes related to pain and attention and cannot be attributed solely to an alteration of the nociceptive system.

### Altered pain processing in patients with irritable bowel syndrome

In patients with IBS, pain-related brain activity (N100, P260, and high-gamma oscillations) and pain perception were not significantly decreased by HNCS. These results are in line with previous studies in which somatic pain inhibition by counter-stimulation was decreased or abolished in patients with IBS [5–10, 55, 56]. In contrast, in the control group, HNCS significantly decreased the N100 and P260 compared with HICS and this effect was significantly greater vs. the corresponding changes in the control session. These results are consistent with previous electrophysiological studies [38, 57–61] and indicate that HNCS inhibits pain-related brain activity after accounting for temporal non-specific changes. Indeed, a strength of the present experimental design is that temporal non-specific effects are measured and controlled for during a control session. This excludes confounding habituation effects from pain inhibition produced by HNCS.

Another strength of the present experimental design is the manipulation of selective attention during the application of counter-stimulation (innocuous and noxious), which allows two things. First, inhibition of pain and pain-related brain activity by selective attention can be measured. Second, the contribution of attention decreased pain inhibition during HNCS can be assessed. Results indicate that patients with IBS show no inhibition of pain-related brain activity by selective attention (distraction). In addition, they show a lack of inhibition (pain and pain-related brain activity) by HNCS when attention is focused either on shock pain or on counter-stimulation. The significant associations between inhibition of pain, N100, and P260 by HNCS and inhibition of pain, N100, and P260 by selective attention support the idea that attentional processes contribute to the inhibition deficit observed in patients with IBS. Consistent with the lack of inhibition by selective attention, patients with IBS reported increased pain vigilance compared with controls. This may indicate that attention is not redirected from one source of pain to another, but rather divided and maintained on both sources at the same time. Consistent with the idea that pain hypervigilance may contribute to the lack of inhibition by HNCS and selective attention, the N100 and P260 inhibition by either intervention was not significant after controlling for individual differences in pain vigilance. This suggests that group differences may be at least partly explained by processes underlying pain vigilance. This is in line with studies suggesting that psychological symptoms may contribute to the development of IBS [62–64] and studies showing increased psychological symptoms in IBS [6, 65–73]. However, the effects of HNCS and selective attention on high-gamma power remained significant after controlling for individual differences in pain vigilance, which suggests that psychological factors cannot explain every aspect of altered pain processing in IBS and that these alterations may develop, at least in part, independently from psychological symptoms.

In addition to event-related potentials (N100 and P260), painful stimuli also elicit ERSPs. With phasic painful stimuli, increased oscillations at low frequencies (1–10 Hz), suppression of alpha and beta oscillations (8–29 Hz), and increased gamma oscillations (30–100 Hz) occur between 150 and 350 ms post-stimulus. These responses have been shown to be modulated by bottom-up and top–down processes [74], and gamma oscillations seem to reflect pain intensity [74]. In the present study, these responses were clearly observed, but only gamma oscillations were modulated by HNCS and selective attention in the control group, while no significant change was produced in patients with IBS. In both groups, shock pain evoked robust high-gamma oscillations generated by sources located in areas related to pain, attention, and cognition. Consistent with the results discussed above, shock-evoked high-gamma oscillations were attenuated in the control group only, in the left lateral prefrontal cortex (including left DLPFC), left premotor regions, medial prefrontal cortex, ACC, and left foot region of primary somatosensory cortex. Inhibition of shock pain responses in these structures during sustained pain further supports their contribution to counter-stimulation analgesia, in line with previous studies [75–82]. In addition, the DLPFC and the ACC are involved in attention and top–down regulation [83–86]. Their modulation during HNCS and distraction may, therefore, reflect changes in these functions to regulate pain in the control group, while this mechanism is altered in patients with IBS.

### Cognitive inhibition in patients with irritable bowel syndrome

In the present study, we assessed whether cognitive inhibition was decreased in patients with IBS and whether this may contribute to altered pain inhibition processes. Reaction times in the naming condition of the Stroop test were not significantly different between groups, indicating that groups were responding equally fast. Although the effects did not reach statistical significance, patients with IBS showed marginally longer reaction times (*p *= 0.054) during the switching condition. To confirm whether cognitive inhibition is decreased in patient with IBS, future studies with larger samples are needed to replicate these findings. Decreased cognitive inhibition in patients with IBS would be consistent with several studies showing that chronic pain is generally associated with impaired response inhibition [87–93]. Previous studies have also reported decreased cognitive flexibility and impaired visuospatial memory in patents with IBS compared with a control group [19, 94]. In patients with IBS, lower cognitive performance may affect how pain symptoms are processed and perceived and may contribute to altered pain inhibition and further increases in chronic pain symptoms. It could be expected that cognitive inhibition may be specifically altered when stimuli or information to be inhibited is related to pain. Indeed, enhanced attentional capture by pain due to attentional bias towards pain-related information has been reported in patients with chronic pain [23, 24]. The present results indicate that even when stimuli and sensory information is unrelated to pain (e.g., words and colors), cognitive inhibition tend to be altered in patients with IBS, suggesting that the deficit may apply to any stimulus modality.

## Limitations

The foremost limitation of this study is the sample size, which is limited to nine participants per group. Therefore, although the main results are consistent with our hypotheses and with previous findings, the lack of group difference for some measures should be interpreted with caution and deserves further investigations. For example, the lack of NFR inhibition by HNCS in the control group may be due to a lack of power and is not consistent with our previous study that included 31 participants [27]. However, the NFR results are consistent with some of our previous studies, in which NFR was not significantly inhibited by HNCS in healthy volunteers [28, 81]. The discrepancies between studies do not seem to rely exclusively on sample size, but also on inter-individual differences and variability. Lastly, it should be noted that we selected female participants only to limit the potential confound of sex differences and variability. This limits the generalizability of the results.

## Conclusion

The present results indicate that brain mechanisms involved in pain inhibition by HNCS and selective attention are altered in patients with IBS. In addition, patients with IBS showed increased pain vigilance, which contributed to the above-mentioned alterations. Altogether, these results indicate that decreased pain inhibition by HNCS in patients with IBS reflects interactions between several brain processes related to pain and attention and cannot be attributed solely to alterations of the nociceptive system.

## Data Availability

The datasets used and analysed during the current study are available from the corresponding author on reasonable request.
